# Carcinogenic effect of adenylosuccinate lyase (ADSL) in prostate cancer development and progression through the cell cycle pathway

**DOI:** 10.1186/s12935-021-02174-6

**Published:** 2021-09-06

**Authors:** Jinling Liao, Qiong Song, Jie Li, Kechen Du, Yang Chen, Chunlin Zou, Zengnan Mo

**Affiliations:** 1 Center for Genomic and Personalized Medicine, Guangxi key Laboratory for Genomic and Personalized Medicine, Guangxi Collaborative Innovation Center for Genomic and Personalized Medicine, Guangxi Medical University, No. 22 Shuangyong Road, Guangxi Zhuang Autonomous Region, Nanning, 530021 China; 2 Key Laboratory of Longevity and Aging-Related Disease of Chinese Ministry of Education, Center for Translational Medicine, School of Preclinical Medicine, Guangxi Medical University, No. 22 Shuangyong Road, Guangxi Zhuang Autonomous Region, Nanning, 530021 China; 3 Department of Urology, The First Affiliated Hospital of Guangxi Medical University, Nanning, 530021 China; 4 The Reproductive Hospital of Guangxi Zhuang Autonomous Region, Nanning, Guangxi 530218 China

**Keywords:** ADSL, Cell cycle, Prostate cancer, Oncogene, Vitro experiment

## Abstract

**Background:**

Prostate cancer (PCa) is still a serious male malignant disease across the world. However, no exact pathogenesis had been explained. Although adenylosuccinate lyase (ADSL) gene was identified to be important in PCa early in 1987, its comprehensive functions for PCa have not been presented.

**Methods:**

The cBioPortal for Cancer Genomics, Oncomine and GEO database were retrieved to investigate the associations between of the *ADSL* gene and PCa. Then, the PC-3, DU145 and C4-2B cell lines were applied in vitro experiments. RNA sequencing and further western blot (WB) were applied to explore the potential mechanisms of ADSL gene in PCa.

**Results:**

Based on PCa clinical datasets, we firstly found ADSL gene highly expressed in PCa tissues. Moreover, its transcript level increased in the metastatic PCa further. Elevated ADSL gene expression indicated a poor prognosis of PCa. While inhibiting the expression of ADSL with siRNA, the ability of cell proliferation and migration all declined markedly, with increased cell apoptosis inversely. Most of cells were blocked in the G0/G1 phase. Additionally, RNA sequencing also discovered the inactivity of cell cycle pathway after ADSL knockdown, which had also confirmed on the proteins levels.

**Conclusions:**

Our study identified the ADSL as an oncogene of PCa through regulating the cell cycle pathway firstly, with explicit cell and clinical phenotypes. Further mechanisms were needed to confirm its carcinogenic effect.

**Supplementary Information:**

The online version contains supplementary material available at 10.1186/s12935-021-02174-6.

## Introduction

Prostate cancer (PCa) is the most common male malignant tumor around the world. In 2018, GLOBOCAN estimated 1.3 million PCa and 359,000 associated deaths worldwide [1]. In United States, the mortality and morbidity of PCa topped the list of all the cancers. Only in 2019, 174,650 new cases and 31,620 deaths were estimated [2]. Furthermore, these data had been increased to 191,930 and 33,330 in 2020 [3]. Although with heavy burden of PCa, exact etiologies including hereditary factors had not been described. Thereinto, adenylosuccinate lyase (*ADSL*) gene had been discovered to have potential role in PCa [4]. *ADSL* is located from 40,346,500 to 40,387,527 bp in chromosome 22 [5]. It is said to be associated with several cancers, including colorectal cancer, breast cancer, melanoma and glioma [4, 6–8]. *ADSL* could enhance cell proliferation, migration, and invasive capability of cancers [9, 10]. Although limited studies had been focused on the association between *ADSL* and PCa risk, upregulated *ADSL* expression was also presented in the PCa. [4] Additionally, *ADSL* mainly participated in the purine metabolism [4]. In PCa, purine metabolism altered prominently, and is still significantly correlated to PSA levels [11]. Further study also confirmed purine metabolism could also influence the PCa cell growth [12]. However, the explicit associations of *ADSL* and PCa had not been explained comprehensively.

## Methods and materials

### Cell culture

Three PCa cell lines (PC-3, DU145 and C4-2B) were cultured in RMPI-1640 Media containing 10% fetal bovine serum (Wisent, Canade), and 1% penicillin/streptomycin antibiotic solution. Another PCa cell line-LNCaP was cultured in RMPI-1640 Media containing 10% fetal bovine serum, 1% GlutaMAX (Life, USA), and 1% Sodium Pyruvate (Life, USA). As the normal prostate cell line, RWPE-1 was grown in keratinocyte serum-free media (Thermo, USA) supplemented with 0.05 mg/ml bovine pituitary extract (SCIENCELL, USA), and 5 ng/ml epidermal growth factor (PeproTech, USA) in 37℃ incubator with 5% CO_2_. All the cell lines were acquired from American Type Culture Collection (Manassas, VA, USA).

### RNA extraction and quantitative real-time PCR (qRT-PCR)

Total mRNA was isolated from cells using RNeasy Mini Kit (Qiagen, Germany), according to the manufacturer’s protocol. RNA was reverse transcribed to cDNA using a Reverse Transcription Kit (Takara, Japan). Quantitative real-time PCR (qRT-PCR) was performed on Roche LightCycler 96 System (Roche, Switzerland), using SYBR Green Master Mix (Roche) with three replicates. The expression of β-actin was treated as reference. The *ADSL* gene primers were 5′-GGAGGCCGAGCAGACATT-3′ in forward, and: 5′-CAGCTTTTGGACAGCAGTGG-3′ as reverse. The β-actin primers were as follows: Forward 5′-CATGTACGTTGCTATCCAGGC-3′; Reverse 5′-CTCCTTAATGTCACGCACGAT-3′.

### Protein isolation and western blotting

Total protein was extracted with RIPA buffer and quantified with bicinchoninic acid (BCA) protein quantitative assay (Thermo, USA). The samples were separated by 10% SDS-PAGE and transferred into PVDF membranes (Merck Millipore, USA). The membranes were blocked in 3% Bovine Serum Albumin (BSA) with PBS. Then, the membranes were incubated with corresponding antibodies: anti-ADSL antibody (Abcam, United Kingdom), anti-β-actin antibody (Cell Signaling Technology, USA), anti-Rb antibody (Cell Signaling Technology), anti-p21 antibody (Cell Signaling Technology), anti-CDK4 antibody (Cell Signaling Technology), anti-CDC2 antibody (Cell Signaling Technology), anti-Bcl2 antibody (Abcam), anti-Bax antibody (Abcam), anti-p27 antibody (Cell Signaling Technology), anti-Bid antibody (Cell Signaling Technology), anti-Bim antibody (Cell Signaling Technology). Appropriate second antibodies were applied in the next incubation. Lastly, the enhanced chemiluminescence (ECL) detection system (ImageQuant LAS 500, USA) was used to detect the membranes following the manufacturer’s protocol.

### siRNA transfection

Three different small interfering RNAs (siRNA) against ADSL were designed [siRNA 2 (sense: CCGAGCAGACAUUGGGUUUTT; antisense: AAACCCAAUGUCUGCUCGGTT); siRNA 3 (sense: CCAACCGACGGAUCUGUUUTT; antisense: AAACAGAUCCGUCGGUUGGTT)]. Then, the most effective siRNA 1 (sense: CCAGUUUCCUGCAGCUCUUTT; antisense: AAGAGCUGCAGGAAACUGGTT) and control siRNA (sense: UUCUCCGAACGUGUCACGUTT; antisense: ACGUGACACGUUCGGAGAATT) (Genechem, Shanghai, China) for *ADSL* was selected for further experiments. 6 × 10^5^ LNCaP and 5 × 10^5^ C4-2B cells per well in 6-well plates, were performed to reverse transfection with 50 nM *ADSL* or negative control siRNA (Genechem) for 72 h using RNAiMAX transfection regent (invitrogen) in Opti-MEM I Reduced Serum Medium (Invitrogen) according to the manufacturer’s protocol.

### Cell growth (MTS) and clone formation

Cell proliferation was evaluated by CellTiter 96 AQueous One solution regent MTS (Promega, USA), according to the manufacturer’s protocol. Briefly, C4-2B and LNcaP cell lines were seeded at 4000 cells/well in 96-well plates. 20ul MTS solution was added to 100ul of RPMI-1640 culture medium and incubated for 4 h in 37 ℃ incubator with 5% CO_2_. Cell viability was evaluated by SYNERGYHT microtiter plate reader (bio-tek) with 490 nm absorbance on day 0, 2, 3 and 4.

Additionally, C4-2B and LNCaP cell lines were seeded respectively at 1000 and 10,000 cell per well in 60mm dishes to assay the clone formation. Visible cell colonies were washed with PBS, and fixed with 4% paraformaldehyde for 30 min. Then, 0.1% crystal violet was used for staining. Finally, colonies in the dish were scanned with scanner and the number of colonies was analysis with Image-J software (National institute of health, Bethesda, MD). All results were repeated for three times.

### Migration

1.5 × 10^5^ C4-2B and LNcaP cells in 200ul serum free medium were placed into the upper side of the transwell chamber (Coning, USA) with a pore size of 8 μm to analyze the cell migration. 600ul containing 10% FBS culture medium was added in the lower well of plate. After incubating for 48 h, the C4-2B and LNcaP cells were fixed with 4% paraformaldehyde for 30 min. 0.1% crystal violet was used for staining. Cells migrating to the basal portion of membrane in the down chamber were counted using microscope.

### Cell cycle

Cell cycle was analyzed with flow cytometry. C4-2B and LNcaP cells were digested and resuspended in precooled 30% PBS and 70% ethanol. Then, they were mobilized for 72 h after siRNA transfection. The stationary cells were recovered by centrifugation with 1800RPM for 5 min. Cells were washed with PBS for 3 times. Then, they were resuspended in 400 µl PI/RNase staining buffer (BD, USA) and incubated 15 min in the dark at indoor temperature. Finally, flow cytometry (BD C6 Plus, USA) was used to detect the cell cycle, which was analyzed by ModFit software (Verity Software House, USA).

In order to confirm the results of cell cycle, EdU staining was also performed in our study. 2× EdU working solution with the complete medium was prepared and added to the C4-2B and LNcaP cells (processed by siRNA for 48 h) in 6-well plate to 10 µM, and incubated for 40 min at 37 °C. Each sample was fixed with 4% formaldehyde, mixed and incubated at room temperature for 15 min. Then, wash the cells twice, centrifuge the tube 5 min at 300 g, and carefully remove and discard the supernatant. Prepare permeabilization solution by adding Triton^®^ X-100 in PBS with the final concentration was 0.25%. Add 100 µl to each sample and incubate for 15 min at room temperature. Prepare click reaction cocktail according to operation manual. Add 500 µl to each tube, mix by pipetting up and down and incubate the tube at room temperature for 30 min without light. Finally, after removing the reaction cocktail and wash cells, 5 µl PI was added to each sample for nuclear stain. Flow cytometric was used to detect the EdU and DNA content.

### Cell apoptosis

C4-2B and LNcaP cell lines were collected from 6-well plate after 72 h siRNA transfection with trypsinization. Then, cells was washed with PBS for 2 times, and resuspended in 1× staining buffer at a concentration of 1–5 × 10^6^ cells per ml. Cell apoptosis was evaluated by AnnexinV-FITC/PI Apoptosis Detection Kit I (BD, USA), according its manufacturer’s protocol. Briefly, the negative control siRNA transfection cells were quadripartition: neither PI nor AnnexinV-FITC, only 5 μl PI stained, only 10ul AnnexinV-FITC stained or both 5 μl PI and 10 μl AnnexinV-FITC. In addition, 5 μl PI and 10 μl AnnexinV-FITC were also stained in the *ADSL* siRNA transfection cells, which were incubated 15 min in the dark on the ice. Cell apoptosis was then detected by flow cytometry (BD C6 Plus, USA) with Cellquest software (BD Biosciences, Franklin Lakes, NJ, USA).

### Statistical data analysis

*ADSL* gene expression analysis was performed in the normal, tumor and metastasis samples with Mann-Whitney U tests or Kruskal–Wallis H test. The clinical PCa datasets were collected from the cBioPortal for Cancer Genomics [13], Oncomine database [14] and GEO database [15]. *P* value < 0.05 was considered to be statistically significant. All statistical analyses were performed in RStudio (version 1.1.453) with R version 3.5.2.

The clinical features including Gleason score (GS), tumor stage and PSA level were collected. Based on GS, the patients were divided into three groups: ≤ 6, 7 and ≥ 8. PSA levels were divided into (4–10], (10–20] and “> 20” (unit ng/ml). The tumor stage was categorized as T1, T2, T3 and T4. Mann-Whitney U test was applied to compare two groups. For more than two groups, the Kruskal-Wallis H test was used. The PCa prognosis and survival were also assessed with the Kaplan Meier survival analysis to evaluate the effects of *ADSL* gene expression in overall survival, biochemical recurrence and metastasis free survival. The patients were divided into two groups based on the median expression of *ADSL* gene expression level. R package “Survival” version 2.40 (https://cran.r-project.org/web/packages/survival/index.html) was used to perform the survival analyses. Statistical analyses for all Kaplan Meier curves were calculated by using log-rank test and cox proportional hazards model to assess the hazard ratio (HR).

### Differentially expressed genes (DEGs) with ribose nucleic acid sequences (RNA-seq)

The *ADSL* silencing (siRNA 1) and corresponding negative control (Control siRNA) LNCap cell lines were sent for the RNA-sEq. Raw reads were loaded in the SOAPnuke (https://github.com/BGI-flexlab/SOAPnuke) for the first filter with the low quality threshold 15, low quality rate threshold 0.2 and N rate threshold 0.05. Then, clean reads were aligned to UCSC hg19 (https://genome-idx.s3.amazonaws.com/hisat/hg19_genome.tar.gz) with the hisat2 (version 2.1.0). FeatureCounts (http://bioinf.wehi.edu.au/featureCounts/) was applied to calculate the gene expression. R package “DESeq2” was further used to acquire the differentially expressed genes (DEGs) after comparing the *ADSL* silencing and control cell lines. The DEGs were visual with the R packages “ggplot” and “pheatmap”.

### KEGG pathway

R package “clusterProfiler” was used to perform the KEGG enrichments. The significant genes (P < 0.05 and |logFC| > 1) were inputted. Top 15 pathways (P < 0.1 and q < 0.2) were shown to explain the gene function.

### Gene set enrichment analysis (GSEA)

GSEA (http://software.broadinstitute.org/gsea/index.jsp) was performed with selected significant DEGs (P < 0.1 and |logFC| > 0.6). The Molecular Signatures Database (MSigDB, ‘c2.cp.kegg.v6.2.symbols.gmt’) was used as the reference. 1000 gene label permutations were used in the calculations. And the adjusted q value < 0.05 was identified as the significantly enriched KEGG pathways.

## Results

### Clinical datasets identified *ADSL* as an oncogene for PCa

**Fig. 1:**
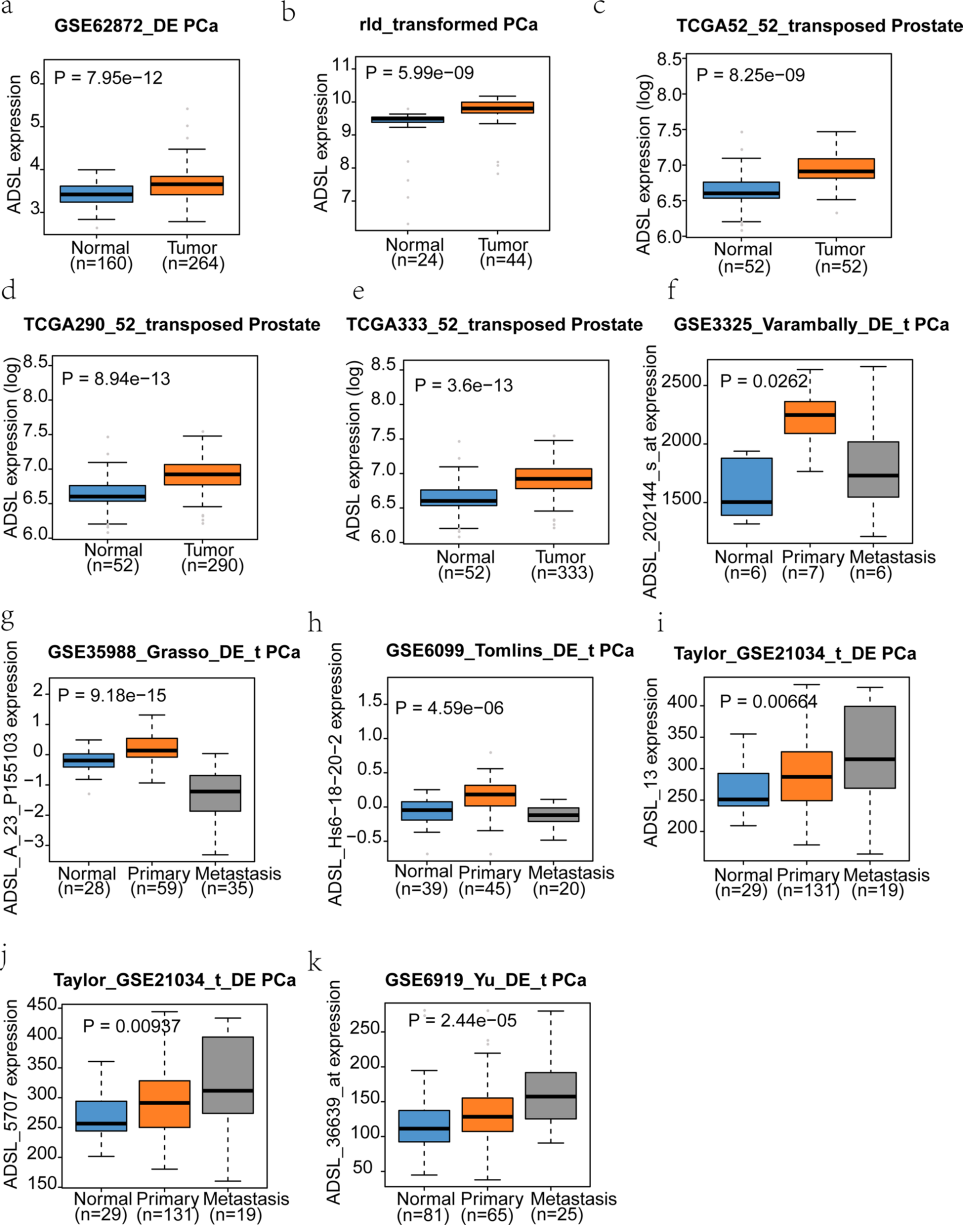
*ADSL* mRNA is strikingly expressed in the human primary prostate cancer and metastatic tissues. **a–h** *ADSL* mRNA is higher in the PCa markedly than normal tissues. **i**–**k** Two datasets (GSE21034 and GSE6919) also discovered upward gradient of *ADSL* transcript levels from normal tissue to metastasis PCa

**Fig. 2:**
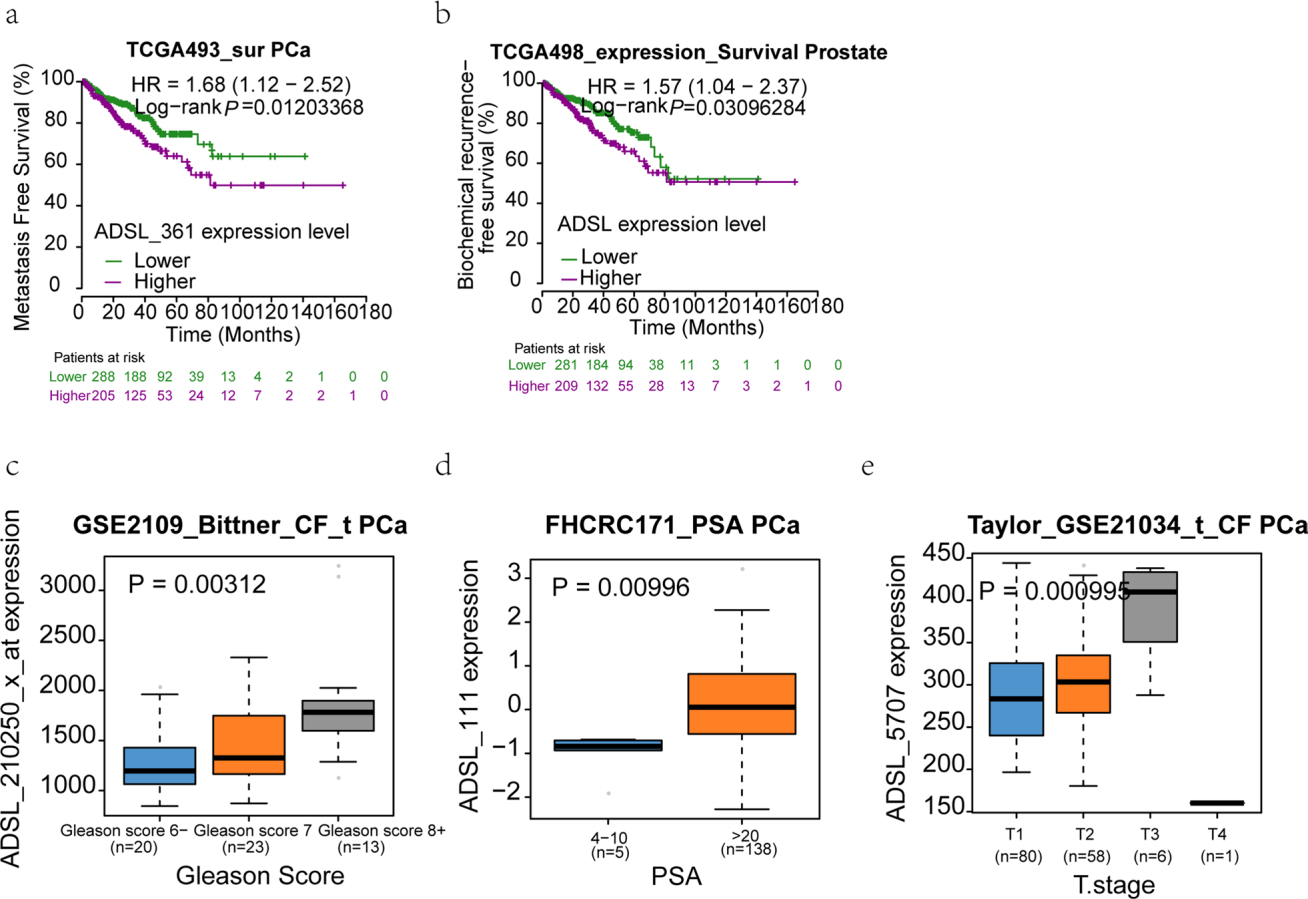
Elevated *ADSL* mRNA resulted in PCa aggression and poor prognosis. **a**, **b** Higher *ADSL* mRNA indicated shorter time for metastasis and biochemical recurrence. **c**–**e** Elevated *ADSL* transcript levels in the aggressive PCa tissues

Base on cBioPortal for Cancer Genomics, Oncomine and GEO database, all available PCa datasets were collected. *ADSL* gene expression was significantly higher in the PCa (P < 0.05) than normal tissues, which hinted the potential carcinogenicity of *ADSL* (Fig. 1). Moreover, two data from GSE21034 and GSE6919 also discover upward gradient of *ADSL* gene expression from normal tissue to metastasis PCa (Fig. 1i–k). When collecting the clinical features (Gleason score, PSA level and tumor stage) and survival data, we excitedly found that high *ADSL* gene expression might shorten the time of metastasis-free survival (HR = 1.68 (1.12–2.52), *P* = 0.012) and biochemical recurrence (HR = 1.57 (1.04–2.37), *P* = 0.003) (Fig. 2a, b). Additionally, along the aggression of PCa, *ADSL* expression also presented the visibly rising trend (Gleason score: *P* = 0.003; PSA level: *P* = 0.010; tumor stage: *P* = 0.001) (Fig. 2c–e).

### *ADSL* promoted human PCa cell proliferation and migration in vitro

**Fig. 3:**
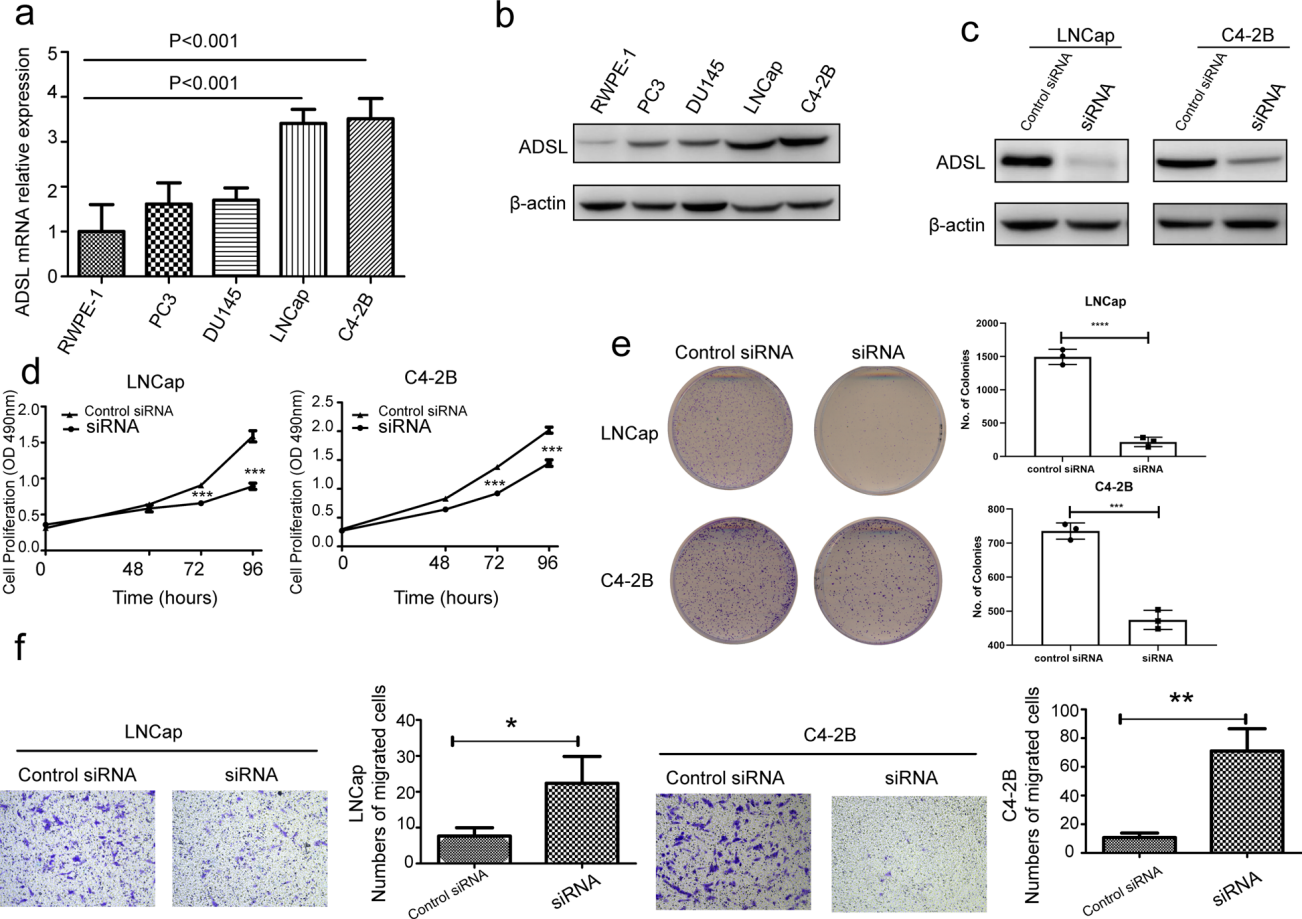
*ADSL* gene level affects prostate cancer cell proliferation and migration in vitro. **a**, **b** The mRNA and protein level of *ADSL* in prostate cancer cell lines. **c** Transfected siADSL in LNCaP and C4-2B cells. **d** MTS proliferation assays evaluated cell proliferation after transfected siADSL in LNCaP and C4-2B cells. **e** Cell proliferation was analyzed with colony formation assays. **f** Cell migration ability were tested by transwell migration assay. *P < 0.05, **P < 0.01, ***P < 0.001 comparing to control siRNA or RWPE-1 cells

The above data indicate that ADSL was a potential oncogene in the PCa occurrence. In order to follow this hypothesis, cell experiments were performed after regulating the *ADSL* mRNA expression. Western blotting and qRT-PCR both hinted highest levels of *ADSL* mRNA and protein in LNCaP and C4-2B PCa cell lines comparing to two other PCa cell lines (PC3 and DU145) and normal prostate cell (RWPE-1) (Fig. 3a, b). Then, *ADSL* was knocked down by siRNA in these two cell lines to evaluate the effect of *ADSL* in cell proliferation and migration in vitro (Fig. 3c). The MTS proliferation and colony formation assays suggested that knockdown *ADSL* gene significantly decreased the proliferation of LNCaP and C4-2B cells compared with negative control cells (Fig. 3d, e). Then, further migration assay also showed that downregulation of ADSL transcript level could also decrease the migration of LNCaP and C4-2B (Fig. 3f).

### *ADSL* affect cell cycle and apoptosis progression

**Fig. 4:**
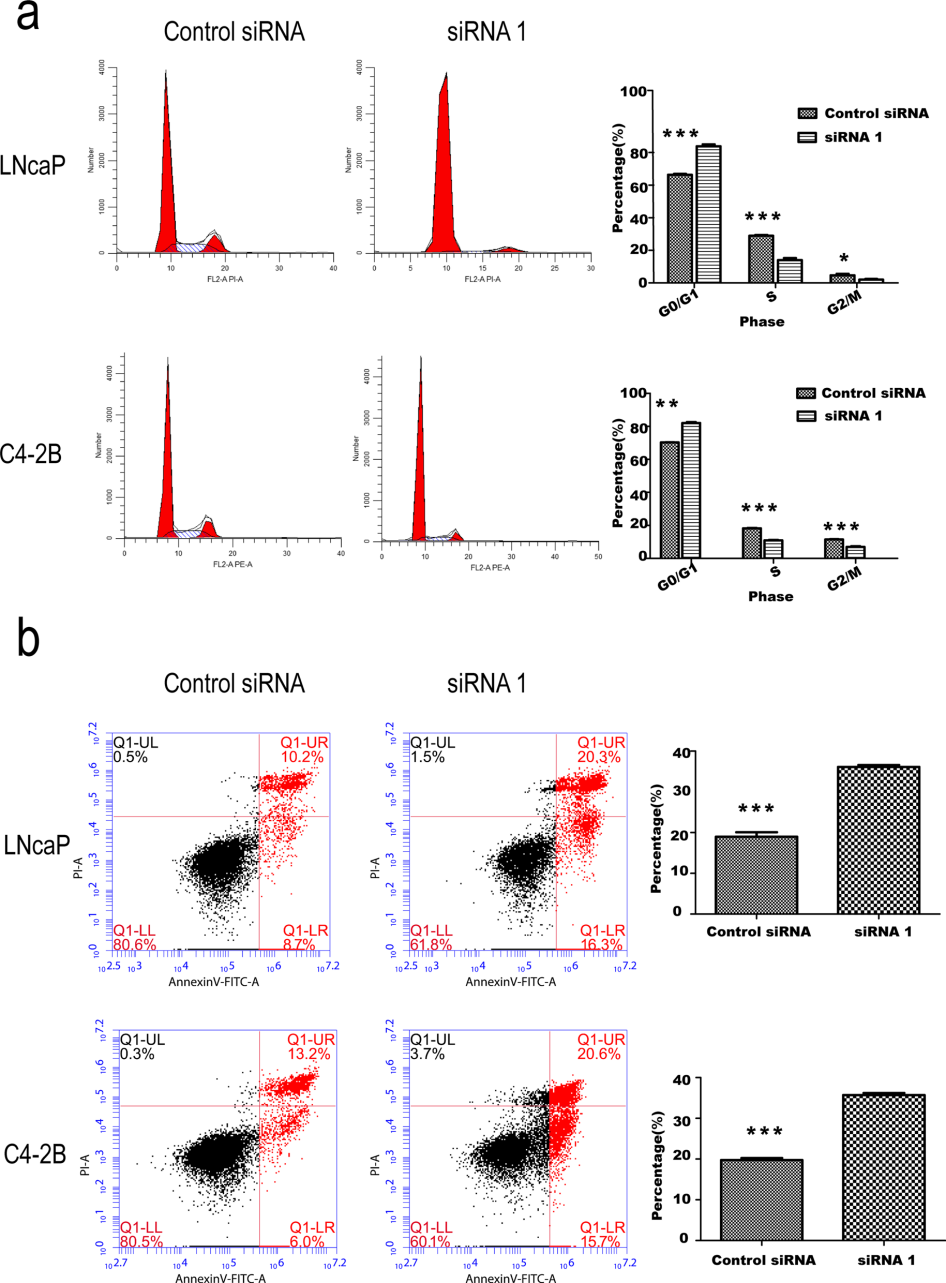
siADSL affect cell cycle and cell apoptosis progression in vitro. **a** Transfected siADSL in LNCaP and C4-2B cells reduce the cell proliferation measured by flow cytometry. **b** And enhances cell apoptosis. *P < 0.05, **P < 0.01, ***P < 0.001 comparing to control siADSL

Previous results suggested the *ADSL* was an oncogene influencing the cell proliferation and migration distinctly. Then, cell cycle analysis was also performed by Flow cytometry in LNCaP and C4-2B cells after silencing the *ADSL* expression. The result showed that siADSL significantly increased the percent of cells in G0/G1 phase by 17.56 and 11.78% for LNCaP and C4-2B cells, respectively. And the percent of S phase (14.92 and 7.24%) and G2/M phase (2.63 and 4.54%) reduced, comparing to control siRNA of LNCaP and C4-2B cells (Fig. 4a). The same results were also confirmed with the EdU staining (Additional file 1: Figure S1). Otherwise, the cell apoptosis was also investigated using AnnexinV-FITC/PI Apoptosis Detection Kit. The result indicated that decline of *ADSL* mRNA could significantly increase the percentage of apoptosis in LNCaP and C4-2B cells to 18.17 and 19.23%, respectively (Fig. 4b).

### *ADSL* promote PCa progression through cell cycle pathway

**Fig. 5:**
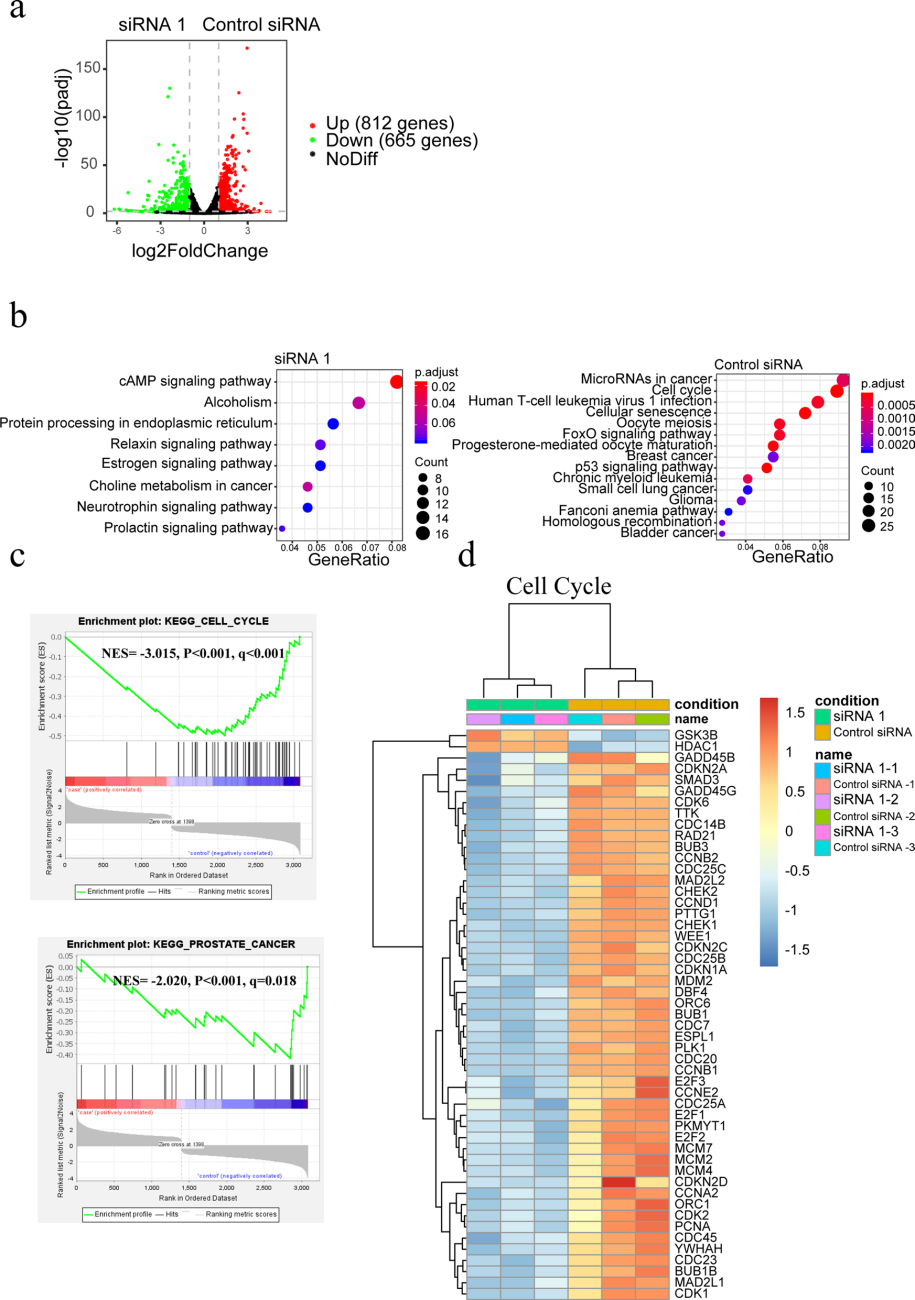
RNA-seq reveals inactivation of cell cycle pathway after *ADSL* knockdown. **a** Volcano plot of differentially expressed genes (DEGs) with 665 up-regulated expression genes after ADSL knockdown. **b** KEGG pathway analysis for the DEGs in the siADSL and control cell lines. **c** GSEA analysis identified cell cycle pathway active in the control cell lines. **d** heat map presented many cell cycle related genes highly expressed in the control groups comparing to siADSL

**Fig. 6:**
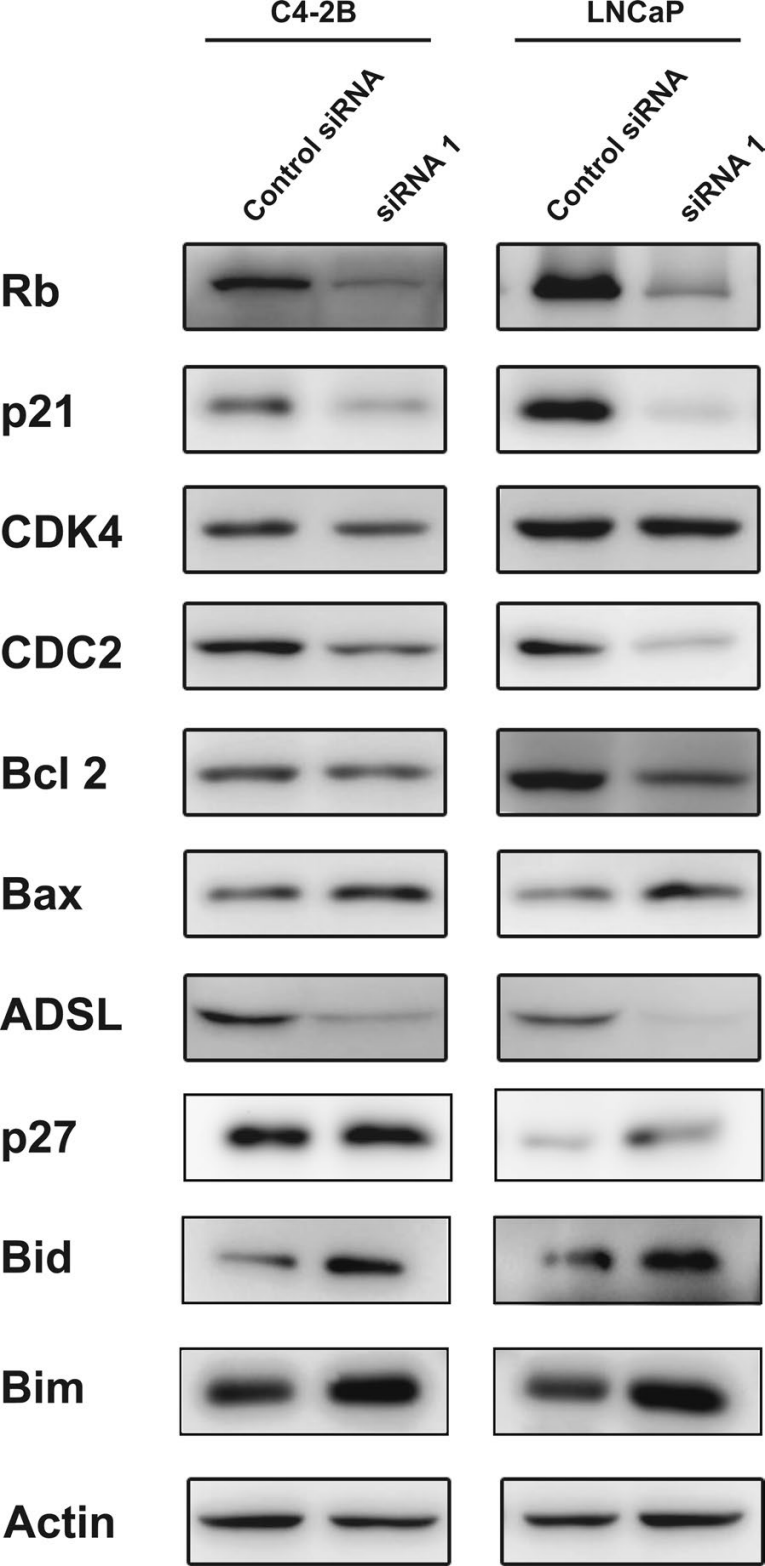
The proteins associate with apoptosis and cell cycle measured by western blotting. *P < 0.05, **P < 0.01, ***P < 0.001 comparing to control siADSL

In this study, *ADSL* gene expression was restrained with designed siRNA. Then, RNA-seq was conducted in the siRNA and negative control cell lines to discuss the potential function of *ADSL* in the PCa development. Comparing to siADSL cells, 812 genes (P < 0.05 and logFC > 1) were up-regulated expression in the control cells, (Fig. 5a) which mainly located in the “MicroRNA in cancer pathway” and “Cell Cycle pathway” (Fig. 5b). GSEA analysis also confirmed the Cell Cycle pathway as the most remarkable compared to siADSL cells (NES = − 3.015, P < 0.001, q < 0.001) (Fig. 5c). Moreover, in the control cells, the prostate cancer pathway also active (NES = − 2.020, P < 0.001, q = 0.018) (Fig. 5c). In the Cell Cycle pathway, many periodic checkpoint genes including Cyclin E1 (*CCNE1*), cyclin dependent kinase (*CDK*) family, and cell division cycle (*CDC*) family highly expressed in the control siRNA cell lines (Fig. 5d). Consistently, the periodic protein expression of retinoblastoma protein (*Rb*), *p21*, *CDK4*, *CDC2* significantly decreased in the siADSL cells. Moreover, down-regulation of anti-apoptosis protein *BCL2* apoptosis regulator (*Bcl-2*) and up-regulation of apoptosis protein *BCL2* associated X, apoptosis regulator (*Bax*), BCL2 like 11 (Bim), and BH3 interacting domain death agonist (Bid) were also presented after restraining the *ADSL* expression (Fig. 6).

## Discussions

PCa is a worldwide disease with ambiguous aetiology. Early in 1987, Reed et al. [4] had firstly discovered the *ADSL* gene might be an indicator of breast and prostate malignancies without comprehensive analysis. In our study, we firstly checked the gene expression of *ADSL* in the normal, tumor and metastasis PCa in all the available datasets. Many datasets presented the higher expression of *ADSL* in the PCa tissue comparing to normal groups. Moreover, two datasets had also found upward gradient of *ADSL* mRNA expression from normal tissue to metastasis PCa, which emphasized the carcinogenic effect of *ADSL* in the PCa development and progression. Survival and clinical analyses also confirmed it as oncogene further. In order to verify these results, cell experiments were performed. After the *ADSL* gene knockdown, the proliferation and migration capability of PCa cell lines were attenuated distinctly. Cells were mainly restrained in G0/G1 stage. Moreover, the cell apoptosis declined in siADSL cells. Preliminarily, we tried to understand the underlying mechanisms of *ADSL* in influencing the PCa. The RNA-seq was performed in the siADSL and control cell lines, which found the Cell Cycle pathway was inactive after *ADSL* gene knockdown. Cell cycle was said to be important in dealing with both endogenous and exogenous sources of DNA damage. Its imbalance would be the critical determinants of cancers [16]. Targeting the cell cycle, some medicines such as β-carboline alkaloids [17], abemaciclib [18], paclitaxel [19] et al., had presented significant therapeutic prospects in cancers. Moreover, some potential anticancer agents including diosmetin [20] and anethole [21] could also inhibit PCa cell proliferation mainly through arresting cell cycle. In 2015, Rubicz et al. draw the landscape of cell cycle genes expressions in PCa, which suggested these genes were associated with a twofold increase in risk of lethal PCa [22]. Many pivotal genes also influence the PCa development through the cell cycle regulation [23, 24]. Lin et al. [25] discovered the ww domain-containing oxidoreductase (WWOX) gene could suppress PCa progression through the cell cycle arrest in G1 phase. Then, in 2017, Yu et al. [26] also identified the critical role of cyclin-dependent kinase inhibitor 3 (CDKN3) in PCa development via cell cycle and DNA replication signaling. In our study, we firstly identified *ADSL* as an oncogene gene in PCa, mainly through cell cycle pathway with some key periodic proteins expression including Rb, p21, p27, CDK4, CDC2 changed. In mammalian, the CDKs and their regulatory subunits participate in the regulation of cell cycle. And it is activated by partial phosphorylation of Rb [27]. In cells, hypophosphorylated Rb could arrest the cell cycle into G0/G1. Inversely, the hyperphosphorylated form induced by the complexes of Cdk4/6-cyclin D, Cdk2-cyclin E and Cdk2-cyclin A could promote the cell cycle progression [28]. After obstructing the ADSL expression, total Rb was down-regulated, which is consistent with previous study [29]. Moreover, low CDK4 expression in the siADSL cells might also further reduce the phosphorylation of Rb and arrest the cell proliferation for influencing the complexes of Cdk4/6-cyclin D. As the family of CDK, CDC2 was known to interact primarily with cyclin B, then participating in the G2-M transition [30]. In the siADSL, low CDC2 expression presented, which suggested ADSL might also restrain the cell G2-M transition. Additionally, as famous CDKs inhibitors, p21 and p27 mainly controlled the cell cycle by regulating CDK activity [31]. Low p27 expression had been proved to promote the PCa development [32, 33]. In the naive PCa cells, p27 was higher expression than siADSL cells, which confirmed the ADSL as a carcinogene of PCa. Although p21 might be an inhibitor of cell cycle, overexpression of p21 also found in the poor PCa progression [34, 35]. The paracrine growth stimulatory effect presented in the p21-induced cells might explain the antiapoptotic and pro-mitogenic effect of p21 [36]. Consistent with our hypothesis, ADSL was a carcinogene. After suppressing ADSL expression, down-regulation of anti-apoptosis protein *BCL2* and up-regulation of apoptosis protein Bax, Bid and Bim were prominent. Above all, ADSL gene could promote the PCa development and progression by controlling the cell cycle gene expressions. Further mechanisms were also under consideration (Additional file 2).

## Conclusions

PCa was a worldwide male malignant tumor with mysterious pathogenesis. Many PCa-related genes had been described. This time, we firstly discovered the potential carcinogenesis of *ADSL* gene in PCa development and progression via cell cycle pathway. Further studies were needed to confirm these effects.

## Supplementary Information

**Additional file 1: Figure S1.** ADSL influence the human prostate cancer cells cycle confirmed with EdU staining by flow cytometry. * *P* < 0.05, ** *P* < 0.01, *** *P* < 0.001 comparing to control siADSL.

**Additional file 2.** The whole original WB blot images with ladder markers in our study.

## Data Availability

The data and materials were in this paper.
