# Supplementary figures and images for: Carcinogenic effect of adenylosuccinate lyase (ADSL) in prostate cancer development and progression through the cell cycle pathway
Source: Cancer Cell Int. 2021 Sep 6;21:467. doi: 10.1186/s12935-021-02174-6 (PMC8419980; doi:10.1186/s12935-021-02174-6)

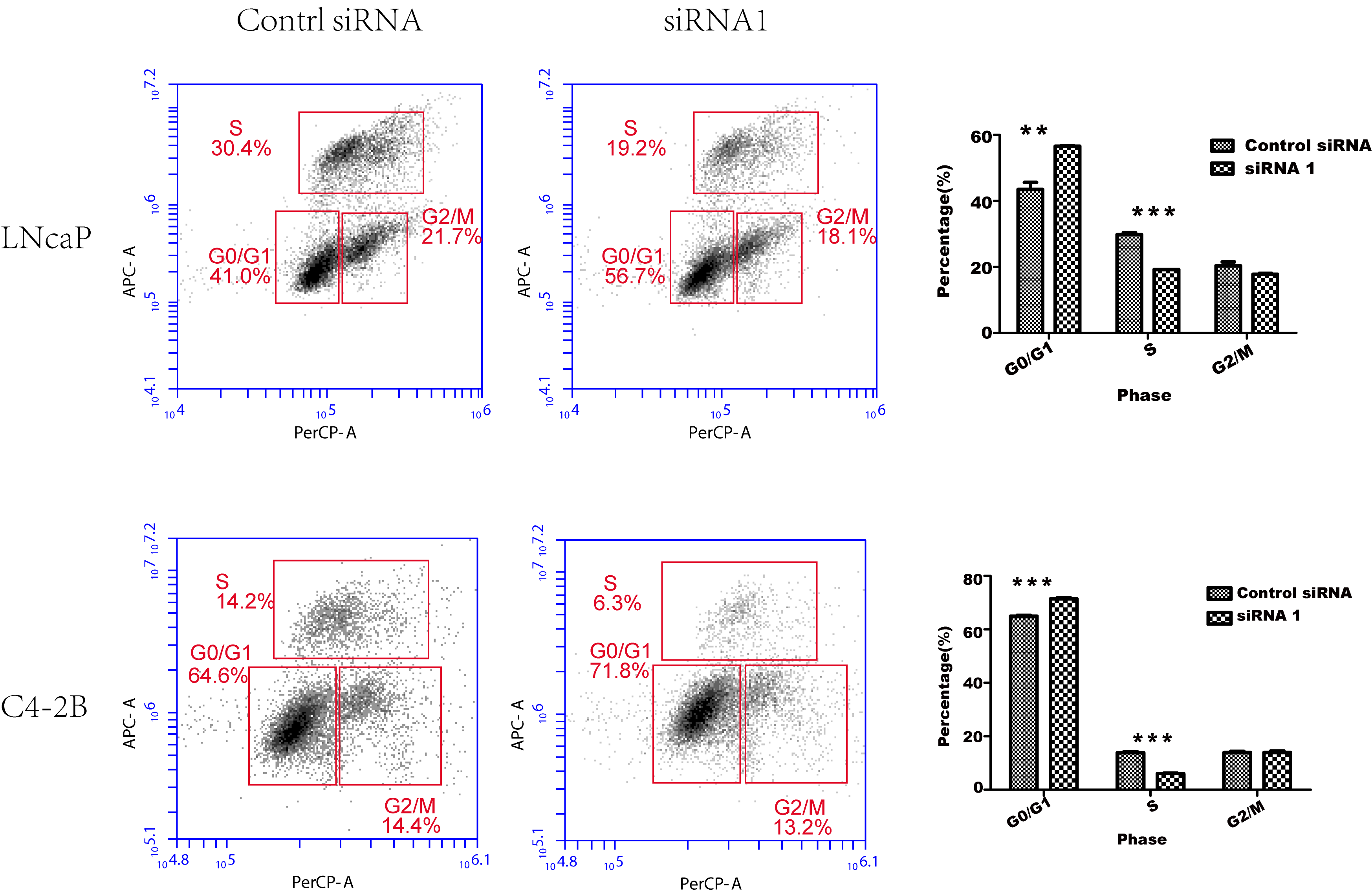

Supplement: Supplementary file 1 — Additional file 1: Figure S1. ADSL influence the human prostate cancer cells cycle confirmed with EdU staining by flow cytometry. * P < 0.05, ** P < 0.01, *** P < 0.001 comparing to control siADSL. [file 12935_2021_2174_MOESM1_ESM.tif]
